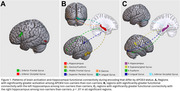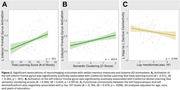# Evidence of Early *APOE4‐*Related Vulnerabilities in Memory Systems in Midlife Women

**DOI:** 10.1002/alz70856_099115

**Published:** 2025-12-24

**Authors:** Katrina A Wugalter, Rebecca C. Thurston, Minjie Wu, M. Ilyas Kamboh, Howard J Aizenstein, Carol A. Derby, Pauline M Maki

**Affiliations:** ^1^ Department of Psychology, University of Illinois Chicago, Chicago, IL, USA; ^2^ Department of Psychiatry, School of Medicine, University of Pittsburgh, Pittsburgh, PA, USA; ^3^ University of Pittsburgh Alzheimer's Disease Research Center, Pittsburgh, PA, USA; ^4^ School of Public Health, University of Pittsburgh, Pittsburgh, PA, USA; ^5^ Department of Neurology, and Department of Epidemiology and Population Health, Albert Einstein College of Medicine, Bronx, NY, USA; ^6^ Departments of Psychiatry, Psychology, and Obstetrics & Gynecology, University of Illinois Chicago, Chicago, IL, USA

## Abstract

**Background:**

Among older adults, *APOE4*‐related associations with neuroimaging outcomes are more pronounced in women than men. At midlife, a critical period for prevention and treatment of risk factors for cognitive decline, the influence of *APOE4* genotype on women's cognitive and brain health is subtle. Therefore, there is a need for early biomarkers of brain vulnerabilities in female *APOE4* carriers. Here, we examined *APOE4‐*related differences in patterns of activation and hippocampal functional connectivity during word encoding in cognitively normal midlife women and the associations of these patterns with verbal memory performance and plasma Alzheimer's disease (AD) biomarkers.

**Method:**

Women participating in MsBrain, a cohort study of brain health in midlife women, completed functional magnetic resonance imaging assessments during verbal encoding and recognition tasks. We measured both activation patterns and hippocampal functional connectivity, the latter using generalized psychophysiological interaction analyses (SPM12, Conn) with AlphaSim (AFNI) to correct for multiple comparisons. *APOE4* group differences (carriers [*E3E4* and *E4E4*] *vs*. non‐carriers [*E3E3*]) were tested using linear regression. Associations of neuroimaging indices with verbal memory (California Verbal Learning Test measures [CVLT learning, semantic clustering]) and plasma AD biomarkers (Aβ42/40, p*‐*tau 181, *p*‐tau 231) were tested via linear regression. All analyses adjusted for age, race, and education.

**Result:**

In 145 women (mean age=59.1 years, 86.2% white, 24.1% *APOE4+*), *APOE4* carriers and non‐carriers did not significantly differ on in‐scanner verbal recognition performance, CVLT measures, or plasma AD biomarkers. During verbal encoding, *APOE4* non‐carriers had significantly greater activation and hippocampal functional connectivity in several regions compared to *APOE4* carriers (Figure 1). Of the regions showing greater activation among non‐carriers, left inferior frontal gyrus activation was positively associated with CVLT measures, and greater connectivity from the left hippocampus to the left declive/fusiform was associated with lower p*‐*tau 181 levels (Figure 2).

**Conclusion:**

Female midlife *APOE4* carriers have decreased activity and connectivity in key memory regions during word encoding compared to non‐carriers, despite showing similar cognitive performance and plasma amyloid and tau levels. Associations of certain connectivity outcomes with AD biomarkers suggest relevance to AD pathogenesis. These functional brain patterns may emerge earlier than the adverse effects of *APOE4* genotype on cognition and brain.